# Use of biologic drugs in juvenile idiopathic arthritis patients followed in an adult rheumatology clinic: real-life data from the HUR-BIO biologic registry

**DOI:** 10.1007/s10067-026-07938-x

**Published:** 2026-01-14

**Authors:** Emine Büşra Ata, Levent Kılıç, Büşra Fırlatan Yazgan, Sevgi Gözde Kart Bayram, Mustafa Ekici, Erdinç Ünaldı, Ali Aytuğ Kuştaş, Buğu Bulat, Ömer Karadağ, Ali Akdoğan, Şule Apraş Bilgen, Sedat Kiraz, İhsan Ertenli, Yelda Bilginer, Seza Özen, Umut Kalyoncu

**Affiliations:** 1 Department of Internal Medicine, Hacettepe University, Ankara, Turkey; 2 Division of Rheumatology, Department of Internal Medicine, Gulhane Training and Research Hospital, University of Health Sciences, Ankara, Turkey; 3 Division of Rheumatology, Department of Internal Medicine, Hacettepe University, Ankara, Turkey; 4 Division of Pediatric Rheumatology, Department of Pediatrics, Hacettepe University, Ankara, Turkey

**Keywords:** Biologic disease modifying anti-rheumatic drugs, Enthesitis related arthritis, Juvenile idiopathic arthritis

## Abstract

**Objectives:**

This study aimed to determine whether there are differences in biologic treatment and complications according to subgroups of juvenile idiopathic arthritis (JIA) patients in adulthood.

**Method:**

HUR-BIO (Hacettepe University Rheumatology Biologic Registry) has been a single-center biologic disease-modifying anti-rheumatic drug registry since 2005. Patients were selected from HUR-BIO who met the International League of Associations for Rheumatology classification criteria for juvenile idiopathic arthritis.

**Results:**

Enthesitis-related arthritis (82, 49.1%), rheumatoid factor (-) polyarthritis (39, 23.4%), and rheumatoid factor ( +) polyarthritis (26, 15.6%) were the most prevalent subgroups in 167 JIA patients. Etanercept (105, 62.9%), adalimumab (28, 16.8%), and infliximab (21, 12.6%) were the most prescribed first-line biologic drugs. Secondary failure was the most common reason for the treatment changes among 42 (43.3%) patients, followed by non-life-threatening side effects in 12 (12.4%) and primary failure in 11 (11.3%). Polyarticular JIA showed higher HAQ (*p* = 0.020) and DAS28-ESR (*p* < 0.001) before biologics and had a longer disease duration to initiation of biologic treatment than enthesitis-related arthritis (*p* < 0.001). Only one patient (1.2%) in the enthesitis-related arthritis group needed the hip prosthesis, while 12 patients (18.5%) in the polyarticular JIA group required the same procedure (*p* < 0.001).

**Conclusions:**

In adult rheumatology departments, the most common JIAs present with ongoing disease are enthesitis-related arthritis and polyarthritis. Secondary failure was the main reason nearly half of the patients needed biologic changes during follow-up. The polyarticular group appears to have a higher disease severity and level of disability.

**Table:** 

**Key Points** • *The most of juvenile idiopathic arthritis patients in adult rheumatology clinics require biologics are enthesitis-related arthritis and polyarticular arthritis.* • *Approximately half of the patients require biologic changes during follow-up, most of these due to secondary failure.* • *Disease severity and disability appear to be higher in the polyarticular group, therefore treatment intensification may be considered.*

## Introduction

Juvenile idiopathic arthritis (JIA) is an umbrella term for childhood rheumatic diseases of unknown etiology that begins before the age of 16, lasts longer than 6 weeks [1]. According to The International League of Associations for Rheumatology (ILAR) diagnostic criteria, there are 7 groups under the group of JIA that differ from each other in terms of both pathogenesis and disease courses [1, 2]. However, it has been noticed that JIA patients are investigated under the same framework in studies and treatment approaches. In the early stages of JIA treatment, a stepwise approach is used, such as non-steroidal anti-inflammatory drugs (NSAID), intra-articular steroid injections, and conventional synthetic disease-modifying antirheumatic drugs (DMARD), while in recent years, the use of biologic DMARDs (bDMARDS) targeting cytokine blockade has increased to reduce the disease's permanent damage and the side effects of systemic steroids [3, 4]. When JIA patients reach adulthood, they are transferred to adult rheumatology clinics through a transitional care process. The European Alliance of Associations for Rheumatology (EULAR)/Pediatric Rheumatology European Association (PReS) standards and recommendations for the transitional care of young people with juvenile-onset rheumatic diseases were published to standardize this transition process [5]. Patient adherence to the transitional care program is crucial for maintaining low disease activity, preventing flares, and reducing disability [6]. Research indicates that the use of biologics has enhanced adherence [7]. This study aims that showing the adult follow-up of JIA patients in our hospital's biological DMARD database and to reveal whether this disease group differs in terms of treatment and complications according to subgroups.

## Materials and methods

### Patients selection and study group

Hacettepe University Rheumatology Biological Registry Database (HURBIO) is a single-center database established in 2005 to monitor drug efficacy and safety and disease follow-up of rheumatology patients receiving biological treatment at Hacettepe University Faculty of Medicine. The HURBIO database consists of three different databases: Spondyloarthritis (SpA) (3448 patients), rheumatoid arthritis (RA) (2365 patients) and psoriatic arthritis (PsA) (634 patients). For our study, a total of 6447 patients who were followed up in our hospital between 2005 and 2022 were scanned from the database, and it was defined that 172 patients were diagnosed before the age of 16.

The patients' medical records, laboratory and radiological findings were examined, and those diagnosed with juvenile idiopathic arthritis according to the ILAR diagnostic criteria were divided into subgroups such as systemic arthritis, oligoarthritis, rheumatoid factor (RF) (-) and RF (+) polyarthritis, PsA, enthesitis-related arthritis (ERA) and undifferentiated arthritis. For systemic arthritis, one or more of the following findings were required: fever lasting at least three days, accompanied by arthritis, erythematous rash, generalized lymph node enlargement, hepatomegaly, splenomegaly, serositis; for oligoarthritis, 4 or fewer joints were involved in the first 6 months of the disease, and the disease did not show the characteristics of other JIA groups. For the polyarticular arthritis, the criteria used were involvement of 5 or more joints in the first 6 months of the disease and not showing the features of other JIA groups. For the ERA group, at least two of the following criteria were required: tenderness or pain in the sacroiliac region, HLA-B27 positivity, arthritis starting after the age of 6 in a male, acute symptomatic anterior uveitis, history of SpA in first-degree relatives, and not following other JIA groups. For PsA, at least two of the following conditions were required along with psoriasis accompanying arthritis, dactylitis, nail findings, and psoriasis in first-degree relatives. Patients who did not meet any of these criteria were classified as having undifferentiated arthritis [1]. After excluding 3 patients with undifferentiated arthritis and 2 patients who were lost to follow-up, 167 (2.6%) patients were included in the study. The patients were then divided into two groups as polyarticular and ERA and their characteristics were compared. For polyarticular arthritis, both RF (+) and RF (-) patients were included in a single group and defined as polyarticular JIA (pJIA).

### Demographic and clinical data

The patients' demographic information was obtained, including age, gender, body mass index (BMI), age of symptom onset, and age at diagnosis. Concomitant conditions included uveitis, familial mediterranean fever (FMF), and knee/hip prosthesis. RF positivity and the presence of HLA-B27 were detected using laboratory records. The presence of sacroiliitis was determined either using modified New York criteria on x-ray or using the ASAS definition of acute sacroiliitis on sacroiliac magnetic resonance imaging [8, 9].

Steroid, conventional synthetic DMARDs (csDMARD), bDMARDs and targeted synthetic DMARD (tsDMARD) used by patients since diagnosis were documented. Conventional synthetic DMARDs included methotrexate, sulfasalazine, hydroxychloroquine, and leflunomide; bDMARDs included tumor necrosis factor inhibitors (TNFi) agents (adalimumab, etanercept, golimumab, certolizumab, infliximab) and other biologic agents (tocilizumab, ustekinumab, secukinumab, rituximab, anakinra, abatacept, and canakinumab) and Janus kinase (JAK) inhibitors included baricitinib and tofacitinib. To simplify the analysis, tofacitinib, baricitinib and other bDMARDs were studied under biologics. The first biologic, as well as any treatment adjustments and dates, were noted.

### Assessment parameters

Disease activities and functions were recorded before starting biologics and at the last follow-up visit. Patients' disease activities were measured using the disease activity score-28 erythrocyte sedimentation rate (DAS28-ESR), patient global assessments with a visual analogue scale (VAS 0–10), and physical activity problems were measured using the health assessment questionnaire (HAQ) score. The DAS28-ESR, patient global, and HAQ scores of patients before commencing biological treatments, at the last follow-up and mean scores were recorded.

### Ethics approval

The Ethics Committee approved the study of Hacettepe University Faculty of Medicine (No. GO 15/788) and was performed in accordance with the ethical standards laid down in the Declaration of Helsinki of 1975/83. All participants gave written informed consent.

### Statistical analysis

In all statistical analyses, the Statistical Package for Social Sciences (SPSS) 24 Windows program was used. Visual (histograms and probability graphs) and analytical methods were used to assess the compliance of variables to the normal distribution. Mean and standard deviation were used to show data that followed a normal distribution. Numerical data were presented as percentages. In independent groups, the independent sample t-test was used to determine the difference in means. Nominal data was analyzed using the Chi-square and Fisher tests. The Kaplan-Maier test was used to determine the duration of drug use. Type-1 error levels of less than 5% were considered statistically significant.

## Results

### Juvenile idiopathic arthritis subgroups

**Fig. 1:**
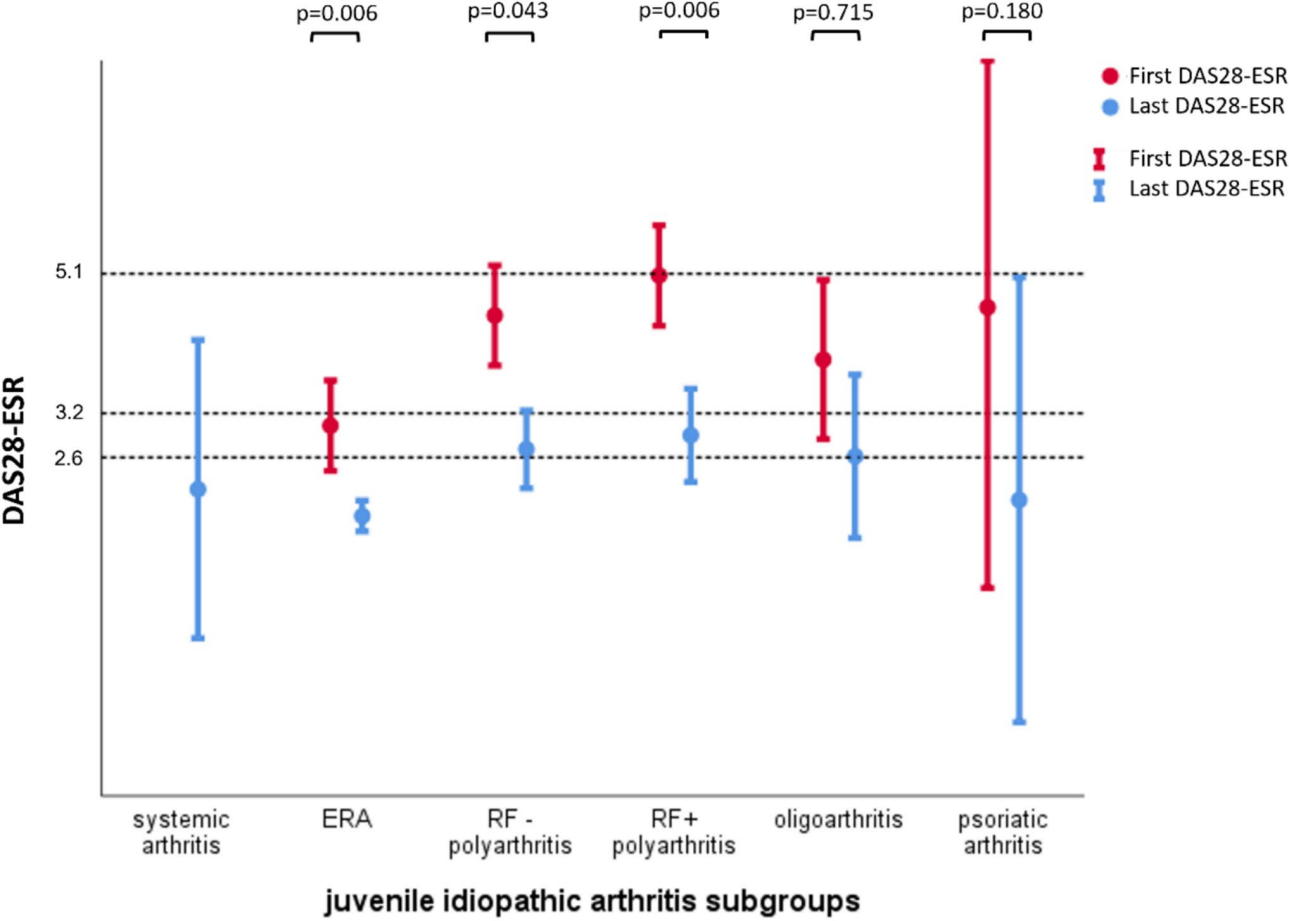
DAS28-ESR values of patients before and after biological treatment according to JIA groups. Figure legend: ERA: enthesitis-related arthritis, ESR: erythrocyte sedimentation rate, RF: rheumatoid factor, DAS: disease activity score, *p* < 0.05 considered statistically significant

At the time of diagnosis, 167 JIA patients were distributed as follows: ERA 82 (49.1%), RF(-) polyarthritis 39 (23.4%), RF(+) polyarthritis 26 (15.6%), oligoarthritis 11 (6.6%), systemic arthritis 6 (3.6%), and PsA 3 (1.8%). Patients with RF (+) polyarthritis and ERA were diagnosed at a later age, but oligoarticular patients were diagnosed at a younger age (*p* < 0.001). In patients with RF (-) polyarthritis, the age at biologic treatment onset and time until biologic treatment were longer (*p* < 0.001) (Table 1). Prior to biologics, disease activity was higher in the polyarticular group compared to ERA and oligoarticular (*p* = 0.004), while HAQ (*p* = 0.359) and VAS global score (*p* = 0.462) were lower in polyarticular group but not statistically significant. Table 1 shows the distribution of patients according to JIA subgroups. All patient groups showed a decrease in DAS28-ESR values following treatment, although some groups did not reach a statistically significant level due to small sample size (Fig. 1).

**Table 1:** Subgroup distribution of JİA patients

	n (%)	Age of symptom onset (year)	Age of diagnosis (year)	Age of biologic treatment onset (year)	Time between diagnosis to biologic treatment	DAS28-ESR before biologic treatment	Mean DAS28-ESR	DAS28-ESR at last visit	HAQ before biologic treatment	Mean HAQ	HAQ at last visit	VAS global before biologic treatment	Mean VAS global	VAS global at last visit
Systemic JIA	6 (3.6)	9.8 (3.9)	10.3 (4.6)	14.8 (4.7)	4.5 (6.5)	-	2.3 (1.0)	2.2 (1.9)	-	0.2 (0.3)	0.3 (0.7)	-	30.8 (16.3)	35.0 (28.8)
ERA	82 (49.1)	11.8 (2.9)	13.6 (2.3)	18.5 (4.8)	5.0 (5.1)	3.0 (1.2)*	1.7 (0.8)*	1.8 (0.9)*	0.6 (0.4)*	0.2 (0.3)*	0.2 (0.3)*	60.6 (22.0)*	29.7 (20.3)	28.0 (24.0)
RF + polyarthritis	26 (15.6)	12.2 (3.2)	12.9 (3.4)	19.5 (7.3)	6.7 (7.7)	5.1 (0.9)*	2.8 (1.4)*	2.9 (1.5)*	1.3 (0.8)*	0.4 (0.4)*	0.3 (0.4)*	70.0 (18.2)	36.7 (26.0)*	37.7 (29.5)
RF—polyarthritis	39 (23.4)	7.7 (4.5)	9.2 (5.0)	23.4 (10.7)	14.2 (10.5)	4.5 (1.4)*	3.0 (1.2)*	2.7 (1.6)*	1.1 (0.9)*	0.6 (0.6)*	0.7 (0.8)*	70.0 (23.3)	42.0 (23.2)*	44.5 (31.1)
Oligoarthritis	11 (6.6)	7.5 (4.6)	7.6 (4.5)	15.6 (4.8)	8.0 (5.9)	3.9 (1.0)*	2.5 (1.7)*	2.6 (1.7)	0.7 (0.8)*	0.3 (0.5)	0.4 (0.7)	45.0 (40.0)	36.8 (28.8)	36.4 (35.3)
Psoriatic arthritis	3 (1.8)	12.0 (3.0)	13.0 (3.6)	27.0 (8.9)	14.0 (6.1)	4.6 (1.3)*	3.4 (0.0)*	2.0 (1.2)	0.6 (0.2)*	0.2 (0.0)	0.1 (0.1)*	60.0 (0)	50.0 (0)*	18.3 (27.5)

DAS28-ESR: disease activity score 28-erythrocyte sedimentation rate, *HAQ* health assessment questionnaire, *VAS* visual analogue scale, JIA: juvenile idiopathic arthritis, *ERA* enthesitis-related arthritis, *RF* rheumatoid factor

*Due to the retrospective design of the study, some data was unavailable. Pre-biological HAQ in 8 ERA patients, 6 RF + polyarthritis patients, 14 RF – polyarthritis patients, 3 oligoarthritis patients, and 2 psoriatic arthritis patients; pre-biological DAS28-ESR in 15 ERA patients, 7 RF + polyarthritis patients, 17 RF – polyarthritis patients, 4 oligoarthritis patients, and 2 psoriatic arthritis patients; pre-biological VAS global in 25 ERA patients, 7 RF + polyarthritis patients, 17 RF – polyarthritis patients, 4 oligoarthritis patients, and 2 psoriatic arthritis patients could be evaluated. Mean HAQ could be evaluated in 34 of ERA patients, 21 of RF + polyarthritis patients, 34 of RF – polyarthritis patients, and one of the psoriatic arthritis patients; mean DAS28-ESR was assessed in 10 of ERA patients, 18 of RF + polyarthritis patients, 31 of RF – polyarthritis patients, 9 of oligoarthritis patients, and one of the psoriatic arthritis patients; and mean VAS global was assessed in 21 of RF + polyarthritis patients, 35 of RF – polyarthritis patients, and one of the psoriatic arthritis patients. HAQ at the last follow-up could be evaluated in 64 of ERA patients, 24 of RF + polyarthritis patients, and 38 of RF – polyarthritis patients; DAS28-ESR at the last follow-up was assessed in 81 of ERA patients, 23 of RF + polyarthritis patients, and 38 of RF – polyarthritis patients; VAS global at the last follow-up could be evaluated in 24 of RF + polyarthritis patients

### Data of biologic treatment

**Fig. 2:**
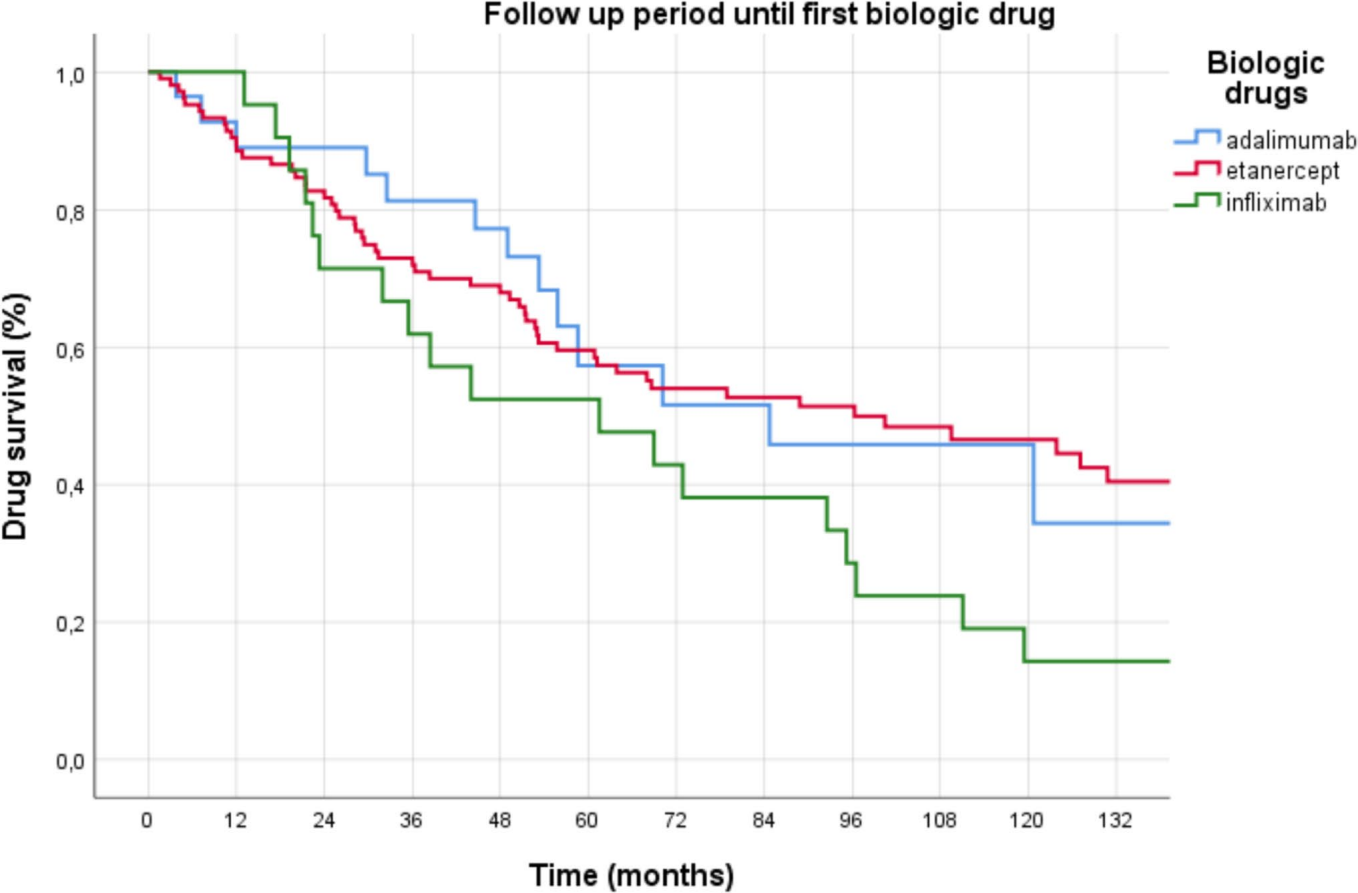
Follow-up time under first biological drug

TNFi was the first choice of biologic drug treatment in 162 (97%) cases, followed by other biologic drug treatments in 5 (3%) patients. Biologic drug changes were observed in 97 (58%) of the patients. The treatments used in the second line were 147 (88%) TNFi, 20 (12%) other biological therapies, and the drugs used at the last follow-up were 135 (81%) TNFi and 32 (19%) other biologic drugs. Of the first-line biologic drugs, etanercept (105, 62.9%), adalimumab (28, 16.8%), and infliximab (21, 12.6%) were the most used. The most often used treatments at the last follow-up were etanercept (57, 34.1%), adalimumab (45, 27.5%), and tocilizumab (18, 10.8%) (Table 2). The first biologic treatment use lasted for 7.1 ± 1.0 (95% CI 5.0–9.1) years. The total follow-up period for the biologic treatment was 9.9 ± 0.6 (95% CI 8.7–11.2) years. Secondary failure was the most common reason for treatment change in 42 (43.3%) patients, followed by primary failure in 11 (11.3%) patients and non-life-threatening side effects such as injection site reaction, rash, and increased infection frequency in 12 (12.4%) individuals. Etanercept had a longer mean follow-up duration (8.0 ± 2.3, 95% CI 3.6–12.5 years) than infliximab (5.1 ± 1.9, 95% CI 1.3–8.9) (*p* = 0.023) and was similar to adalimumab (7.1 ± 2.5, 95% CI 2.1–12.0) (*p* = 0.865) (Fig. 2). If patients were classified by the presence of swollen joints at last visit, 146 (87.4%) patients had zero swollen joints. These patients’ biologics were mostly etanercept (53, 36.3%), adalimumab (40, 27.4%), tocilizumab (13, 8.9%), and certolizumab (10, 6.8%). If patients were classified according to disease activity, 108 (64.7%) patients were in remission (DAS28-ESR < 2.6). These patients’ biologics were mostly etanercept (40, 37%), adalimumab (30, 27.8%), tocilizumab (11, 10.2%), and infliximab (9, 8.3%).

**Table 2:** Biological drug using data

	Initial biologic treatment *n* = 167	First biologic switch *n* = 97	2 or more biologic switches *n* = 36
**Etanercept**	105 (62.9)	14 (14.4)	5 (13.9)
**Adalimumab**	28 (16.8)	36 (37.1)	6 (16.7)
**Infliximab**	21 (12.6)	13 (13.4)	3 (8.3)
**Certolizumab**	5 (3.0)	8 (8.2)	5 (13.9)
**Golimumab**	3 (1.8)	8 (8.2)	1 (2.8)
**Abatacept**	1 (0.6)	3 (3.1)	0 (0)
**Tocilizumab**	1 (0.6)	11 (11.3)	7 (19.4)
**Anakinra**	3 (1.8)	1 (1.0)	1 (0.6)
**Tofacitinib**	0 (0)	2 (2.1)	2 (5.6)
**Secukinumab**	0 (0)	0 (0)	3 (8.3)
**Canakinumab**	0 (0)	1 (1.0)	1 (2.8)
**Rituximab**	0 (0)	0 (0)	0 (0)
Others*	0 (0)	0	2 (5.6)

*1 Ustekinumab, 1 baricitinib

### Comparison between polyarticular and ERA groups

Patients were divided into two groups: pJIA and ERA. A comparison was made between 82 ERA patients and 65 pJIA patients. Most of pJIA patients were female (52 (80%)), and the age at diagnosis was lower than in ERA (*p* < 0.001). Prior to biologics, the patient global assessments were similar (*p* = 0.284), but pJIA patients had higher HAQ (*p* = 0.020) and DAS28-ESR (*p* < 0.001) scores. The time until biologic treatment was longer in pJIA than in ERA (*p* < 0.001) (Table 3).

**Table 3:** Demographic and clinical data of patients with enthesitis-related arthritis and polyarticular arthritis

		ERA (82)	Polyarticular arthritis (65)	*p*
**Demographic and clinical findings**	Age (years)	29.0 ± 6.1	32.9 ± 9.5	**0.006**
	Gender (female)	14 (17.1)	52 (80.0)	** < 0.001**
	Age of symptom onset (years)	11.8 ± 2.9	9.5 ± 4.6	**0.001**
	Age of diagnosis (years)	13.6 ± 2.3	10.7 ± 4.7	** < 0.001**
	Time from symptom to diagnosis (years)	1.8 ± 2.4	1.2 ± 2.0	0.105
	Mean duration of disease (years)	15.5 ± 6.5	22.2 ± 10.4	** < 0.001**
	Body Mass Index kg/m^2^	23.2 ± 4.3	22.1 ± 4.3	0.118
	Familial Mediterranean fever	24 (29.3)	6 (9.2)	0.074
	Uveitis	15 (18.3)	11 (16.9)	0.845
	Hip replacement	1 (1.2)	12 (18.5)	** < 0.001**
	Knee replacement	0 (0)	3 (4.6)	0.084
**Treatment modalities**	Methotrexate use (ever)	47 (57.3)	58 (89.2)	** < 0.001**
	Methotrexate use (with biologics)	3 (3.7)	15 (23.1)	** < 0.001**
	Leflunomid use (ever)	9 (11.0)	20 (30.8)	**0.003**
	Leflunomid use (with biologics)	0 (0)	12 (18.5)	** < 0.001**
	Sulfasalazine use (ever)	66 (80.5)	35 (53.8)	**0.001**
	Sulfasalazine use (with biologics)	0 (0)	6 (9.2)	**0.007**
	Steroid use (ever)	36 (43.9)	54 (83.1)	** < 0.001**
	Steroid use (with biologics)	1 (1.2)	27 (41.5)	** < 0.001**
	Age at biologic start (years)	18.5 ± 4.8	21.9 ± 9.6	**0.012**
	Time to biologic treatment (years)	5.0 ± 5.1	11.2 ± 10.1	** < 0.001**
	Follow-up period under biologics (years)	7.2 ± 4.7	8.0 ± 5.2	0.365
	Initial biologic (etanercept)	46 (56.1)	47 (72.3)	**0.043**
**Pre-biological evaluation**	Pre-biologic HAQ*	0.6 ± 0.4	1.2 ± 0.8	**0.020**
	Pre-biologic VAS global*	60.6 ± 22.2	67.3 ± 21.6	0.284
	Pre-biologic DAS28-ESR*	3.0 ± 1.2	4.7 ± 1.3	** < 0.001**
**Biological treatment response**	Last control HAQ*	0.2 ± 0.3	0.6 ± 0.7	** < 0.001**
	Last control DAS28-ESR*	1.8 ± 0.9	2.8 ± 1.6	** < 0.001**
	Last control VAS global*	20.0 ± 24.0	40.0 ± 30.4	**0.007**
	Any biologic switch	39 (47.6)	45 (69.2)	**0.008**
	Mean time to biologic change (years)	6.6 ± 4.4	5.8 ± 4.8	0.409

*ERA* Enthesitis-related arthritis, *HAQ* health assessment questionnaire, *VAS* visual analog scale, *DAS* disease activity score

Data are given as mean ± standard deviation or number (percentage). Bold values indicate statistical significance (*p* < 0.05)

*Pre-biological HAQ; could be evaluated in 8 of ERA patients, 20 of polyarticular JIA patients, Pre-biological DAS28-ESR; 15 of ERA patients, 24 of polyarticular JIA patients, pre-biological VAS global; 25 of ERA patients, 24 of polyarticular JIA patients, HAQ at last follow-up; 61 of ERA patients, 62 of polyarticular JIA patients, DAS28-ESR at last follow-up; 81 of ERA patients, 61 of polyarticular JIA patients, VAS global at last follow-up; all ERA patients, and 63 of polyarticular JIA patients

In ERA, the first biologic medication used was etanercept 46 (56%), followed by infliximab 15 (18%) and adalimumab 15 (18%), but in pJIA, it was etanercept 47 (72%), adalimumab 9 (14%), and infliximab 5 (8%). In the ERA group, further biologic options were certolizumab, anakinra, and golimumab, while abatacept, tocilizumab, and certolizumab were used in pJIA patients. When the biologic drugs used at the last follow-up were examined, etanercept remained the most used biologics in both groups, followed by adalimumab 23 (28%) and infliximab 10 (12%) in ERA patients, and adalimumab 17 (26%) and tocilizumab 13 (20%) in pJIA patients.

At the last follow-up, patients with pJIA had higher scores for VAS global assessment (*p* = 0.007), DAS28-ESR (*p* < 0.001), and HAQ (*p* < 0.001) assessments. In the ERA group, only one patient (1.2%) needed a hip prosthesis, whereas in the pJIA group, 12 patients (18.5%) required (*p* < 0.001) (Table 3). HAQ, DAS28-ESR, and VAS global evaluation scores were compared prior to biological treatment and at last follow-up. After treatment, both groups experienced significant decreases in HAQ [ERA patients 0.6 to 0.2 (*p* = 0.024), pJİA patients 1.2 to 0.6 (*p* = 0.032)], in DAS28-ESR [ERA patients 3.0 to 1.8 (*p* = 0.003), pJIA patients 4.7 to 2.8 (*p* < 0.001)], and in VAS global assessment scores [ERA patients 60.6 to 20.0 (*p* < 0.001), pJIA patients 67.3 to 40.0 (*p* = 0.024)].

## Discussion

This is the first comprehensive study to compare the long-term follow-up of pJIA and ERA, with long follow-up periods under biological therapy. The purpose of this study was to assess the follow-up in adulthood, of JIA patients treated with biological DMARDs to guide rheumatologists in their clinical practice; we have assessed the duration of biologics, comorbidities according to subgroups, activity scores, and disability indices. It is challenging to predict the course and outcome of the disease at the time of diagnosis because it may vary throughout each of the JIA groups. In adulthood, one-third of patients still have arthritis complaints, and it is controversial how to treat patients who have reached adulthood [10].

JIA is the umbrella term for chronic inflammatory arthritis in children [11]. According to the ILAR diagnostic criteria, JIA is classified into seven groups based on pathophysiology and disease progression [1]. Recently pediatricians were revisiting this classification and attempted to use similar terms as adult rheumatology for some subgroups [12]. For example the RF (+) polyarticular JIA observed in adolescent girls, which constitutes only 10% of our JIA patients, may be regarded as an early onset adult RA. On the other hand, recently Still’s disease was suggested to replace sJIA [13]

The most prevalent disorders in our study cohort were ERA (82, 49%) and polyarthritis (65, 39%). This may be because ERA is more frequent in Turkish patients when compared to European JIA patients. When the patients were divided into two groups (ERA and pJIA), the time to biologic initiation and follow-up was longer in pJIA, and DAS28-ESR, HAQ, and patient global assessment scores at the last follow-up and prosthesis rate were higher.

According to epidemiological studies conducted in our country and around the world, oligoarthritis is the most common JIA subgroup [14–16], which is the case in Turkish JIA studies as well. The current study included 11 cases of oligoarthritis (6.6%). This low percentage in the adult cohort is because this is a childhood disease that typically responds to NSAIDs and intra-articular injections, except for the ocular involvement. In a study of JIA children treated with biologicals at our hospital’s pediatric rheumatology department, the complete remission percentage was greatest in oligoarthritis [17]. In the Portuguese cohort that aimed to monitor the long-term effects of biological treatment, while oligoarticular JIA was less common, polyarticular, ERA, and extended-oligoarticular were the most prevalent types[18]. Similar to our analysis, ERA and polyarticular JIA were the two most common groups in a study examining adult JIAs in the biological treatment [19].

After the diagnosis, the goal is to minimize side effects and achieve either full remission or minimum disease activity quickly as possible. Step-up therapy is the standard treatment approach, nonsteroidal anti-inflammatory drugs (NSAIDs) and intra-articular steroid injections are the first-line treatments. In situations where this medication is insufficient, patients might be treated with conventional synthetic DMARDs, followed by bDMARDs [3, 20]. In the current study, the most used biologic drugs were etanercept (most used), adalimumab, and infliximab. These findings are in line with other pediatric reports [17, 18]. 58% of the patients experienced at least one biological change, with secondary failure being the most common reason for treatment change. Similar studies have revealed that 22.5%−40% of individuals experienced biologic switches [17–19, 21]. The present study has one of the longest follow-up periods (9.9 ± 0.6), which contributed to the significant biological change rate. The initial biological follow-up time, which is 7.1 ± 1.0 years, is similar to previous research [18].

There is only limited information on how the JIA subgroups will proceed in adults based on subgroups. This study aims to compare the two most common subgroups we see in adulthood, ERA and pJIA, to investigate if there are differences in parameters such as disease activity, prosthesis use, and responsiveness to biological treatments. While DAS28-ESR and HAQ scores were lower in ERA patients before beginning biological treatment, patient global assessment score was similar to those in pJIA. Following the biological treatment, it was found that both groups had reduced HAQ, DAS28-ESR, and patient global assessment scores. The DAS28-ESR score, which evaluates tender and swollen joints and is an expected finding in pJIA, may not be an objective criterion to evaluate disease activity in ERA. However, the fact that patient global assessment and HAQ scores are greater than ERA may indicate that disease activity is higher in pJIA [22]. This may also be due to fact that we have included RF (+) pJIA patients along with the typical childhood RF (-) pJIA patients in the assessment. The current study found that joint surgery was performed more frequently in pJIA. Since the disease’s duration was longer, and biologic treatment was initiated later, patients' prosthetic requirements and functionality may be influenced by this situation. In a study investigating the effect of time from diagnosis to the start of biologic drugs on the long-term prognosis of JIA, JIA patients were divided into three groups based on time to biologic. HAQ, active disease rate, and joint surgery were found to be higher in the group that started biologics after more than 5 years [23].

There were limitations in our study. Due to the retrospective design of the study, some clinical and laboratory data are missing although most of the missing data are among the pre-biological period. Because the groups compared are not homogeneous, the biological age of onset and duration of disease are not similar. Since joint swelling and tenderness were assessed in DAS28-ESR, it is expected that the polyarticular group will have higher scores. As commented above, we have pooled both the childhood RF (-) polyarticular JIA patients with the RF (+) adolescent polyarticular JIA patients in the pJIA group since the numbers would be small if we had separated them.

In adult rheumatology clinics, ERA and polyarthritis are the most common JIAs that present with ongoing disease. During follow-up, more than half of the patients have required a biologic change, with secondary failure being the most common reason. Furthermore, the severity of the disease and impairment level appear to be higher in the polyarticular group. However, more extensive prospective studies are needed to understand better how JIA progresses in adulthood and how its course varies across different subgroup.

## Data Availability

The datasets generated and/or analyzed during the current study are available from the corresponding author on reasonable request.
